# The Possible Mechanism of Idiosyncratic Lapatinib-Induced Liver Injury in Patients Carrying Human Leukocyte Antigen-DRB1*07:01

**DOI:** 10.1371/journal.pone.0130928

**Published:** 2015-06-22

**Authors:** Makoto Hirasawa, Katsunobu Hagihara, Noriko Okudaira, Takashi Izumi

**Affiliations:** Drug Metabolism & Pharmacokinetics Research Laboratories, Daiichi Sankyo Co., Ltd., Tokyo, Japan; University of Rhode Island, UNITED STATES

## Abstract

Idiosyncratic lapatinib-induced liver injury has been reported to be associated with human leukocyte antigen (HLA)-DRB1*07:01. In order to investigate its mechanism, interaction of lapatinib with HLA-DRB1*07:01 and its ligand peptide derived from tetanus toxoid, has been evaluated *in vitro*. Here we show that lapatinib enhances binding of the ligand peptide to HLA-DRB1*07:01. Furthermore *in silico* molecular dynamics analysis revealed that lapatinib could change the β chain helix in the HLA-DRB1*07:01 specifically to form a tightly closed binding groove structure and modify a large part of the binding groove. These results indicate that lapatinib affects the ligand binding to HLA-DRB1*07:01 and idiosyncratic lapatinib-induced liver injury might be triggered by this mechanism. This is the first report showing that the clinically available drug can enhance the binding of ligand peptide to HLA class II molecules *in vitro* and *in silico*.

## Introduction

Lapatinib is an orally active dual tyrosine kinase inhibitor which interrupts the human epidermal growth factor receptor 2 (HER2) and epidermal growth factor receptor pathways approved for the treatment of HER2-positive advanced or metastatic breast cancer. It is used in combination with capecitabine for patients who have received previous therapy including an anthracycline, a taxane and trastuzumab, and in combination with letrozole for the treatment of postmenopausal women with hormone receptor–positive breast cancer [1,2]. Although lapatinib has an acceptable safety profile in clinical settings, grade 3 alanine aminotransferase (ALT) elevation and serious liver injury with hyperbilirubinemia have been reported in 1.6% and 0.2%, respectively [3]. A retrospective case–control pharmacogenetic study of ALT elevation in lapatinib-treated breast cancer patients revealed a strong genetic association between elevated ALT and the HLA alleles DQA1*02:01 and DRB1*07:01, suggesting a possibility of immune-mediated pathogenesis [4,5]. Although the expressions of these two alleles are highly correlated, comparisons of HLA-DR and HLA-DQ heterodimer protein pairing suggested the HLA-DRB1*07:01 allele provided the simplest causal association with lapatinib-induced ALT elevation [6]. This allele is also associated with ALT elevation during long-term treatment with the oral direct thrombin inhibitor ximelagatran [7]. A competitive binding assay revealed that ximelagatran was able to preferentially compete for binding with a known high affinity ligand peptide of the DRB1*07:01, supporting the specific involvement of DRB1*07:01 allele in idiosyncratic ximelagatran-induced hepatotoxicity. In this study, we evaluated the effect of lapatinib on the binding of the known ligand peptides to HLA-DRB1*07:01 and DRB1*15:01 *in vitro*. Interestingly, lapatinib preferentially increased rather than competed the binding of ligand peptide derived from tetanus toxoid (TT) to HLA-DRB1*07:01 in a dose-dependent manner. Also we performed a series of *in silico* molecular modeling for HLA-DRB1*07:01 and two other HLA-DRs to further assess the possible role of lapatinib on HLA-peptide binding, which could lead to the mechanism of immune-mediated lapatinib-induced liver injury.

## Materials and Methods

### HLA class II binding assay

The binding assay was conducted in EpiVax, Inc. (Providence, RI, USA) as previously reported with minor modifications [8]. Briefly, soluble HLA molecules (2.5 nM) were incubated with increasing concentrations of drugs (0.25–250 μM for lapatinib and lumiracoxib and 2.5–2500 μM for amoxicillin) and biotinylated control ligand peptides (TT 830–844: QYIKANSKFIGITEL for HLA-DRB1*07:01 and myelin basic protein (MBP) 94–112: NPVVHFFKNIVTPRTPPPS for DRB1*15:01; 25 nM) for 24 hours at 37°C to reach equilibrium. HLA-peptide complexes were then captured in triplicate on a 96-well EIA/RIA plate (Corning, #3361) coated with L243 anti-HLA-DRA antibody (BioXCell, #BE0160). The plates were washed and incubated with Europium-labeled streptavidin (Perkin-Elmer, #1244–360) for 1 hour at room temperature. The plates were washed again and DELFIA enhancement solution (Perkin-Elmer, #4001–0010) was added to develop the plates for 15–20 minutes at room temperature before they were read on a SpectraMax M5 plate reader.

### Statistical analysis

Two-tailed Dunnett tests were carried out using SAS System Release 9.2 (SAS Institute Inc.) to compare the Europium fluorescence counts in the presence of drug with that in the absence of drug. A *P* value of less than 0.05 was considered as a significant difference.

### HLA structures utilized for docking and molecular dynamics (MD) simulations

Table 1 summarizes the HLA structures used for both docking and MD simulations. High-resolution experimental x-ray crystallographic structures were available and utilized for DRB1*01:01 and DRB1*15:01, and a homology model of DRB1*07:01 was generated from two different chain templates highly homologous to the DRB1*07:01 sequence, along with the common chain from 1AQD.

**Table 1:** Structures of HLA Proteins Utilized.

Allele	Receptor Structure (pdb chains)
DRB1*01:01	1AQD (A, B)
DRB1*07:01	Modeled on 1BX2 (B); 1AQD (A)
DRB1*15:01	1BX2 (A, B)

### Docking protocol

**Fig 1:**
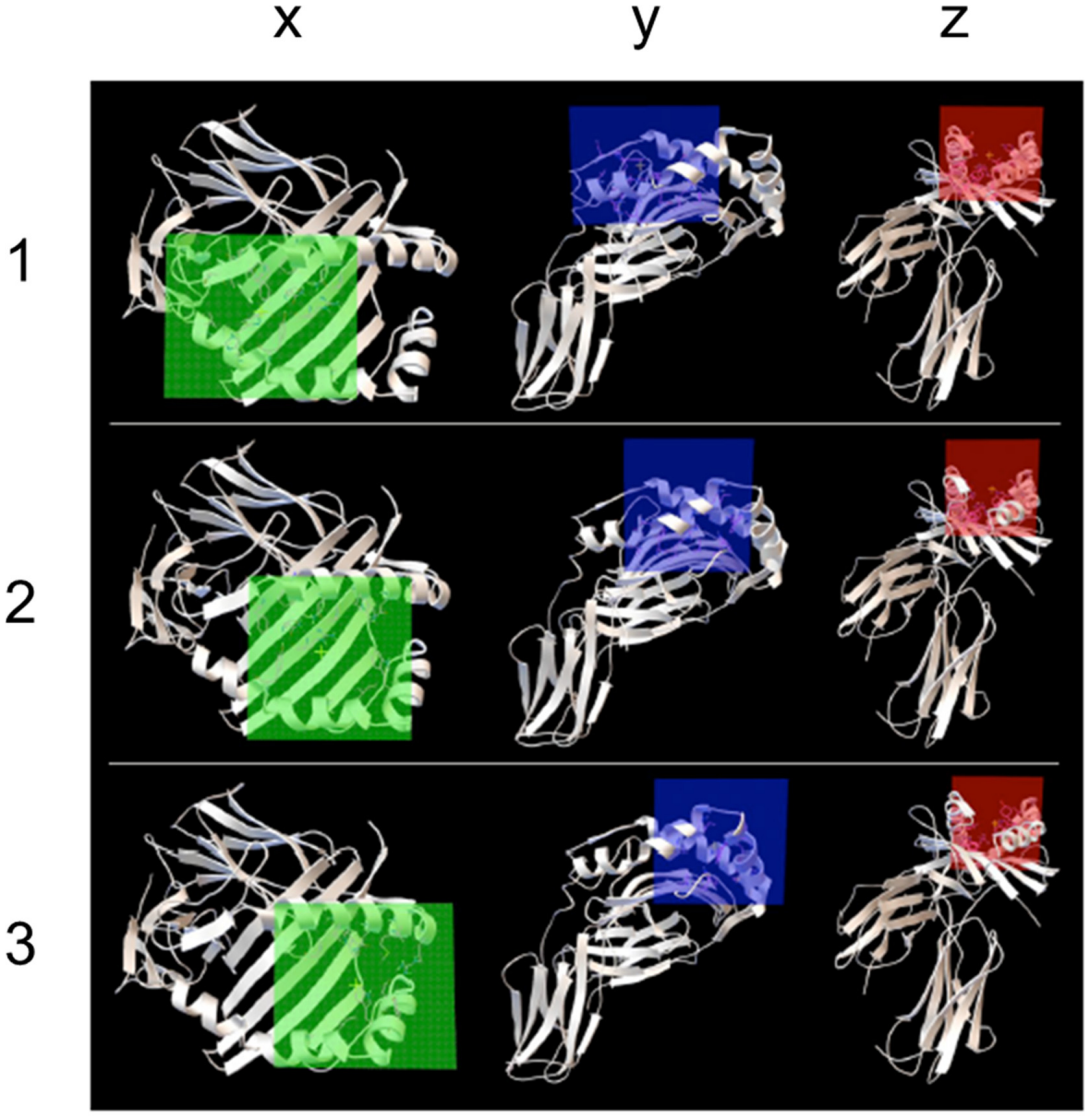
Orthogonal (x, y, z) views of the three docking volumes of the HLA binding groove. (1) Left part (2) middle part (3) right part of the binding groove. Volume extents are shown in green, blue, and red. Each volume was used in a separate docking run and the results were merged.

**Fig 2:**
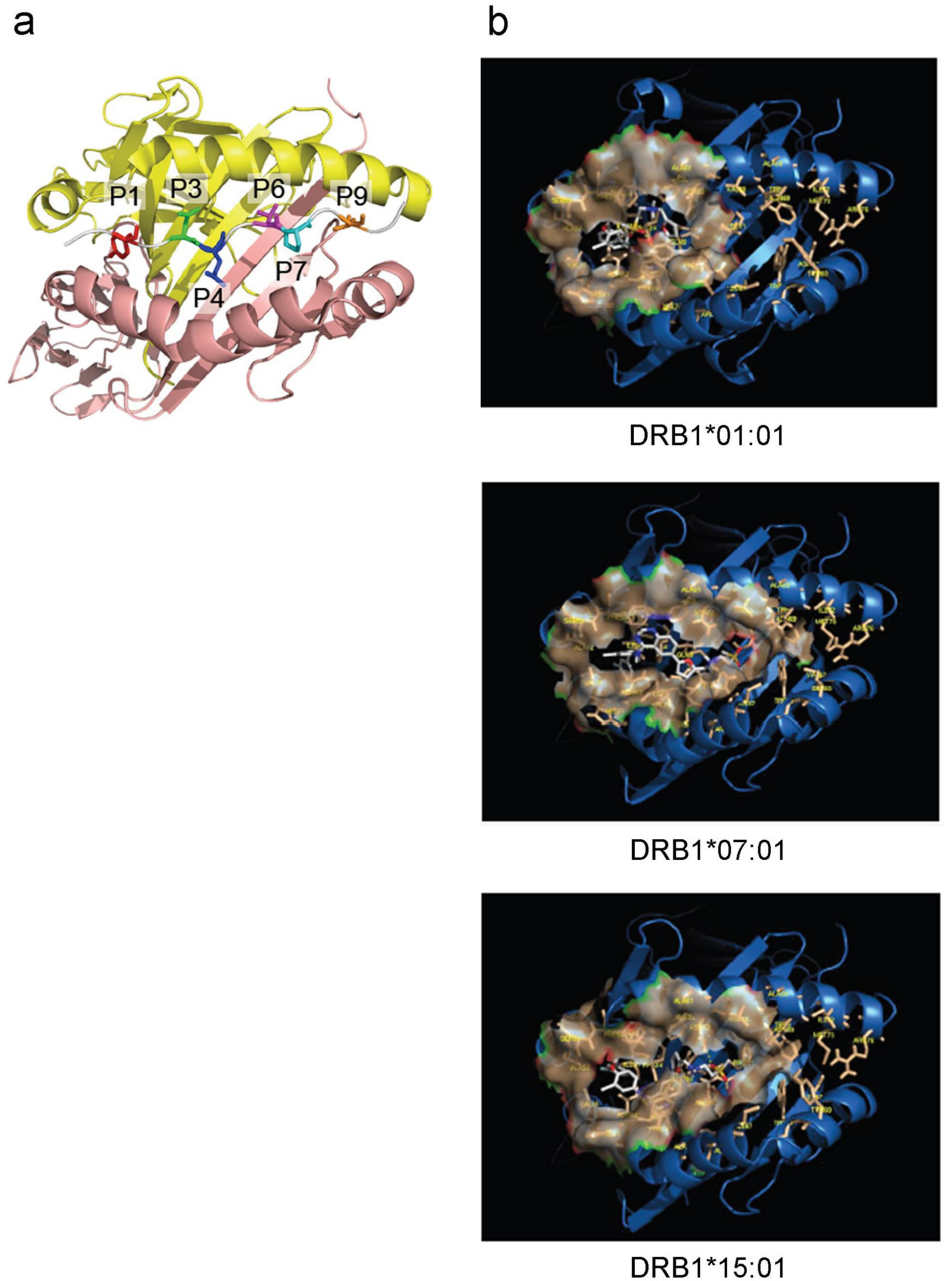
Binding pockets and molecular docking results. (a) Primary binding pockets are illustrated with a representative HLA system. P1 (red), P3 (green), P4 (blue), P6 (purple), P7 (cyan), P9 (orange). (b) Molecular docking results. The top binding mode is illustrated for each HLA-ligand system.

HLA protein structures were prepared for docking according to a standard protocol involving removal of water and ions and protonation at physiological pH. Lapatinib was similarly prepared for docking, with rotatable bonds identified using the prepare ligand4.py script in AutoDockTools package [9]. For computational efficiency, the elongated HLA binding groove was divided into three overlapping volumes covering the full length of the groove (Fig 1); each volume was used in a separate docking run and the results were merged. For each binding volume, the side-chains of residues extending into the volume were modeled as flexible. The structure of lapatinib was obtained from DrugBank (http://www.drugbank.ca/) and the CHARMM and MMFF force fields were used to parameterize lapatinib. Docking was performed using AutoDock Vina [10] with DRB1*01:01, *07:01 and *15:01. The top scoring binding modes across all three search volumes (Fig 2) were selected for the MD simulations described below. Lapatinib was predicted to lie across the bottom of the binding groove interacting with pockets P1-P4 for DRB1*01:01 and P1-P6 for DRB1*07:01 and DRB1*15:01.

### Molecular dynamics simulations

Each system was protonated at pH 7.4 and then solvated in a periodic rectangular box of TIP3P water with 10 Å padding between the edge of the box and the nearest solute atom. Sodium ions were added to neutralize the system. The system was relaxed and then gradually heated to 300 K. The production simulations were performed in the canonical NVT ensemble, which simply ensures that the system maintains constant temperature and volume. An integration time step of 4 fs was used. The ‘representative structure’ of each simulation is selected as the conformation which is the most representative of the conformations assumed over the final 1,000 frames of the simulation.

### MD simulations of HLA molecules (apo structures)

The apo simulations were 10 ns long each. The decreased average slope of the inter-helical distances indicates a closing motion of the binding groove. This is consistent with the idea that HLA moves to a conformation that is no longer receptive for a ligand peptide [11]. Although the size of the binding groove increased somewhat for the DRB1*07:01 over the first 10 ns, an additional 10 ns simulation confirmed that it eventually closed.

### Homology modeling of peptides

For *in silico* analysis, a peptide derived from the influenza virus, haemagglutinin (HA 306–318), was used for DRB1*01:01 in addition to TT peptide for DRB1*07:01 and MBP peptide for DRB1*15:01. The structures of the peptides were obtained through homology modeling (Table 2). The HA peptide shares significant sequence similarity with the peptide of experimental structure 1DLH. Therefore the structure for HA peptide was generated via an *in silico* mutation (lysine to arginine) of PDB 1DLH, chain C. Because there are no suitable PDB structures for the TT peptide, the peptide structure was created *de novo* using the Molefacture Protein Builder utility in the VMD software. Finally, the MBP structure was generated by aligning the structures of the peptides from PDBs 1BX2 and 1FV1, which share a common 12-residue subsequence. The position of the N-terminal asparagine from 1BX2 was grafted onto the structure of the peptide in 1FV1 to produce a model of the full sequence.

**Table 2:** Homology Modeled Peptides.

Name	Target Sequence	Template Sequence	Template PDB ID (chain)
FLU-HA	P**R**YVKQNTLKLAT	P**K**YVKQNTLKLAT	1DLH (C)
TET-TOX	QYIKANSKFIGITEL	N/A	N/A
MBP	**N**PVVHFFKNIVTPRTPPPS	ENPVVHFFKNIVTP;	1BX2 (C);
		PVVHFFKNIVTPRTPPPS	1FV1 (C)

Altered residues are in boldface.

### MD simulations of HLA molecules in the presence of ligand peptides

For each allele, a series of peptide registration frames was considered as the starting positions. The following frames were considered, DRB1*01:01 frames 2, **3**, 4; DRB1*07:01 frames 1, 2, 3; DRB1*15:01 frames 2, **3**, 4, 7. EpiMatrix predicted registrations are indicated with an underline and the frames consistent with experimental x-ray crystallographic structures are indicated in bold. The peptide-bound simulations were each 5 ns long. The most likely peptide binding registrations were selected for each allele according to the energetic stability, contact with α and β chains and consistency with the experimental x-ray crystal structure and the EpiMatrix predicted registration. Based on these analyses, the following peptide registrations were selected to perform the next simulations. For DRB1*01:01, frames 2 and 3 were chosen, and for DRB1*07:01 and for DRB1*15:01, frame 3 were chosen.

### MD simulations of lapatinib-bound HLA molecules in the presence of ligand peptides

The structures of the trimers (HLA-peptide-lapatinib) were modeled by structurally aligning the representative structures from MD simulations of the HLA-peptide and lapatinib-HLA simulations. Small vertical adjustments (typically 3–4 Å) were made to eliminate steric clashes between the peptide and lapatinib. This small shift was quickly resolved and the peptide snapped back to binding to HLA soon after the simulation started.

## Results

### HLA Class II binding assay

**Fig 3:**
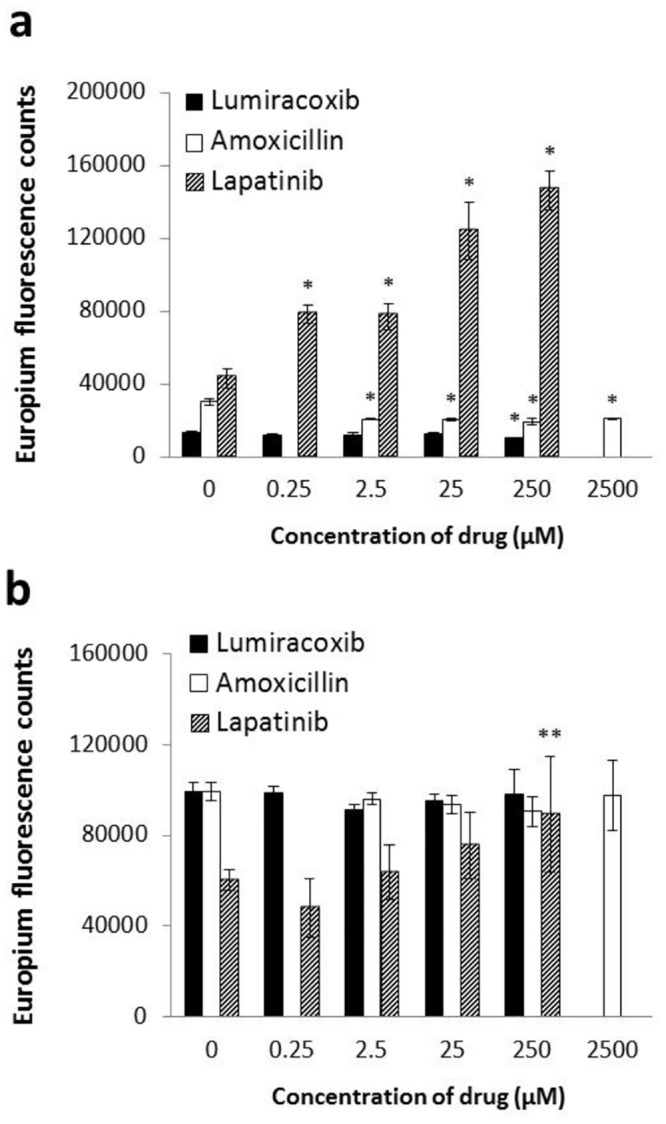
Binding assay results. The binding of ligand peptides to HLA-DRB1*07:01 (a) and DRB1*15:01 (b) after 24 h of incubation in the absence and presence of three drugs, lapatinib, amoxicillin and lumiracoxib are shown. Plots show mean ± s.d. of triplicates. *P < 0.01; **P<0.05 (compared with counts in the absence of each drug).

To evaluate the effects of drugs on HLA-peptide interactions, DELFIA-based binding assay was conducted. The binding assay system was verified by competition of the unlabeled positive and negative control peptides against biotinylated ligand peptides. The positive control peptides successfully inhibited the binding of ligand peptides, but the negative control peptides did not (data not shown). Then, we evaluated the effects of three drugs, lapatinib, that is clinically related to DRB1*07:01 specific liver injury, and amoxicillin and lumiracoxib, that are not related to it, on the binding of its ligand peptide to each HLA-DR (DRB1*07:01 as the test allele and DRB1*15:01 as the control allele). Interestingly, lapatinib increased TT peptide binding to HLA-DRB1*07:01 in a dose-dependent manner, whereas amoxicillin and lumiracoxib did not exhibit increment of peptide binding for both alleles. These results indicate the observed effect is lapatinib specific (Fig 3). The enhancing effects of lapatinib were statistically significant (*P* <0.05) for DRB1*07:01 at 100 times lower concentrations than for DRB1*15:01, which indicates the allele preference of lapatinib for DRB1*07:01 over DRB1*15:01. Lapatinib did not exhibit any autofluorescence in this assay condition. Lapatinib did not show a tendency to affect the affinity of ligand peptide (data not shown), which is consistent with the previous report where addition of abacavir did not shift the IC50 of ligand peptides [12]. Amoxicillin and lumiracoxib decreased the binding of ligand peptide to HLA-DRB1*07:01 significantly, but their effects were not in dose-dependent manners.

### MD simulations of lapatinib-bound HLA molecules in the absence of ligand peptides

**Fig 4:**
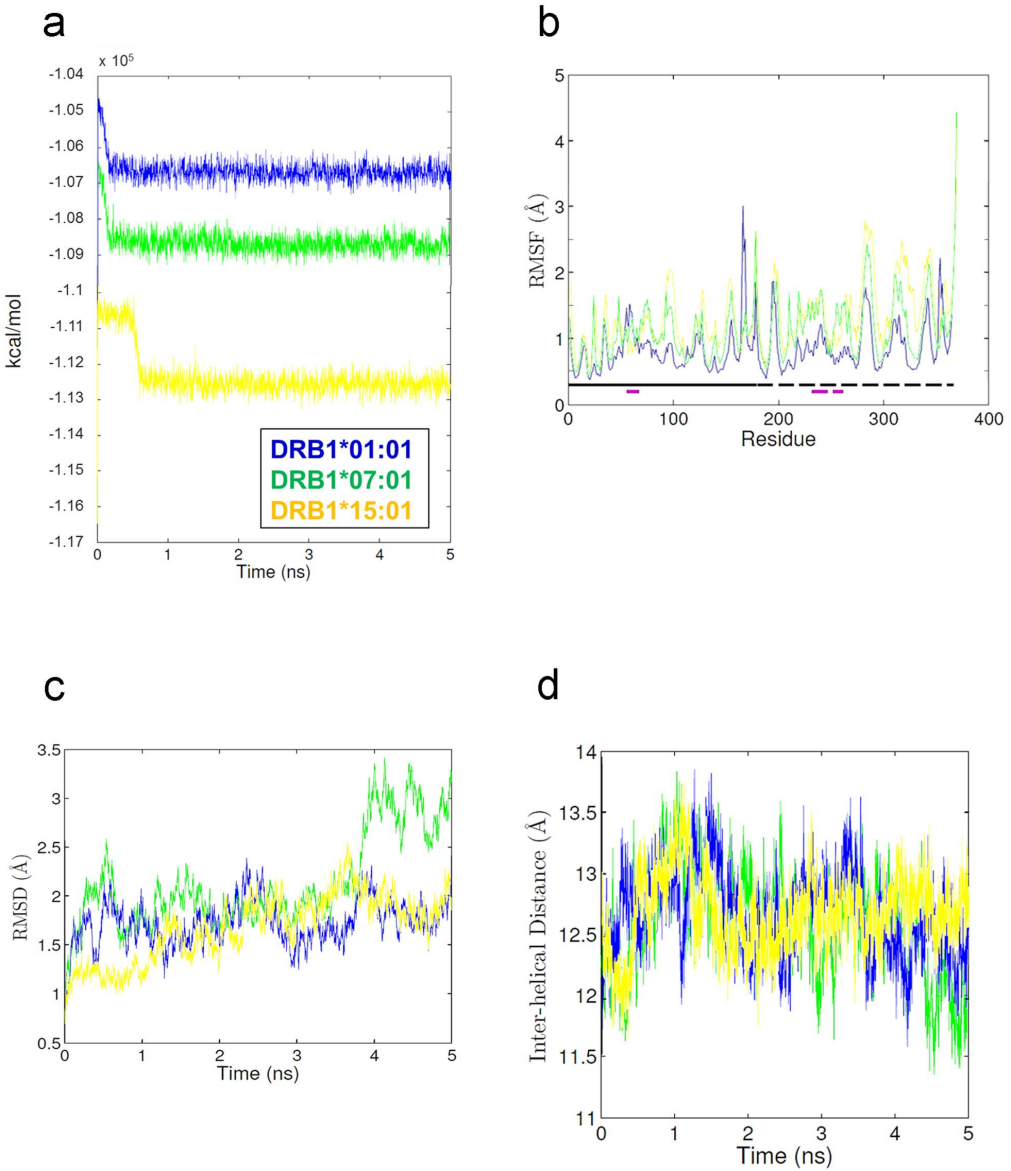
Simulated parameters of lapatinib-bound HLA system. (a) Calculated Energies vs. time plot. (b) RMSF values of polypeptide backbone. The location of α and β chains and the residues that comprise the binding groove helices are indicated by the solid and dashed lines running just above the x-axis. Alpha chain (solid black), alpha chain helix (solid purple), beta chain (dashed black), beta chain helix (dashed purple). (c) RMSD values of polypeptide backbone vs. time plot. (d) The average distance between each Cα in the helix of the α chain and the closest Cα atom in the helix from the β chain.

**Fig 5:**
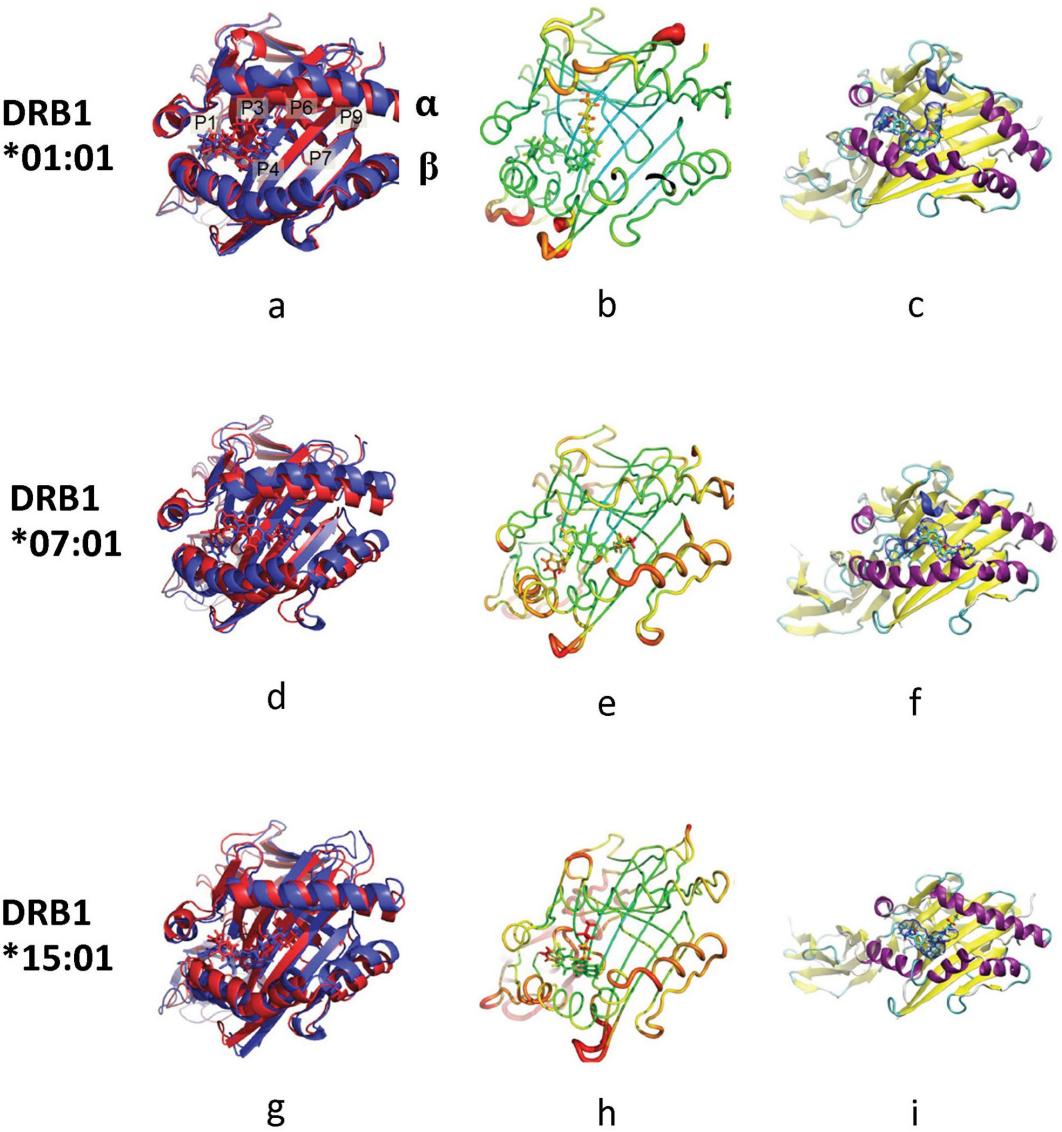
Simulated structures of lapatinib-bound HLA system. Lapatinib-bound HLA-DRB1*01:01 (a-c), DRB1*07:01 (d-f) and DRB1*15:01 (g-i). (a), (d) and (g) Alignment of an initial structure (red) and a representative structure (blue). The difference between the red and blue structures illustrates how the system changes from the beginning of the simulation to the end of the simulation. (b), (e) and (h) Sausage plot of the structure where the color and the thickness of HLA are proportional to the RMSF of α carbon. The color scales for the sausage plots are the same throughout this document. (c), (f) and (i) The volume occupied by lapatinib. The blue envelope means the region occupied by lapatinib at least half the MD frames. Specifically, the blue envelope around lapatinib is the region occupied by lapatinib in at least 50% of the frames of the simulation.

In all simulations, lapatinib remained bound in the binding groove throughout the entire simulation. Each of the energies of lapatinib-bound simulations stabilized within about 0.5 ns (Fig 4a), indicating that the simulations are energetically stable. The flexibility of HLA was very similar in all simulations (Fig 4b). The presence of a lapatinib decreased the overall flexibility of the DRB1*01:01 and DRB1*07:01 alleles compared to the apo simulations. Fig 4c shows that the RMSDs stabilize after 1 to 2 ns, indicating that the systems relaxed quickly. Fig 4d shows that the size of the binding groove changed differently in each system, suggesting that different lapatinib-allele combinations behave differently. In Fig 5 structural analyses of the lapatinib-bound simulations are presented in three panels. The primary differences in three simulations are the binding pose of lapatinib and the degree to which it alters the β chain helix.

In the lapatinib-DRB1*01:01 system, lapatinib binds stably within the binding groove making good contacts in P1 and P4. The sulfonyl end of lapatinib is unbound but stable. Bound lapatinib has a minimal impact on the configuration of the DRB1*01:01 α and β chains (Fig 5a–5c). In the lapatinib-DRB1*07:01 system, lapatinib is relatively stationary except for a small flexible part buried in the binding groove and it appears to sprawl out across pocket P1-P6. The β chain of HLA moves to a more closed position to stabilize the interaction with lapatinib. Given that the binding groove can close in the apo form, the closing around lapatinib indicates a stable interaction and suggests that lapatinib does not interfere with the flexibility of the binding groove (Fig 5d–5f). In the lapatinib-DRB1*15:01 system, lapatinib does not shift locations from initial, but both ends of the ligand are flexible and sitting atop P1-P6. The sulfonyl end is unbound but relatively stable. Although the α chain moves more than other systems, helix integrity is maintained. On the other hand, the β chain is disrupted in this system (Fig 5g–5i). These simulations make a stronger case for lapatinib-DRB1*01:01 and lapatinib-DRB1*07:01 systems than the lapatinib-DRB1*15:01 system in the absence of ligand peptides.

### MD simulations of lapatinib-bound HLA molecules in the presence of ligand peptides

**Fig 6:**
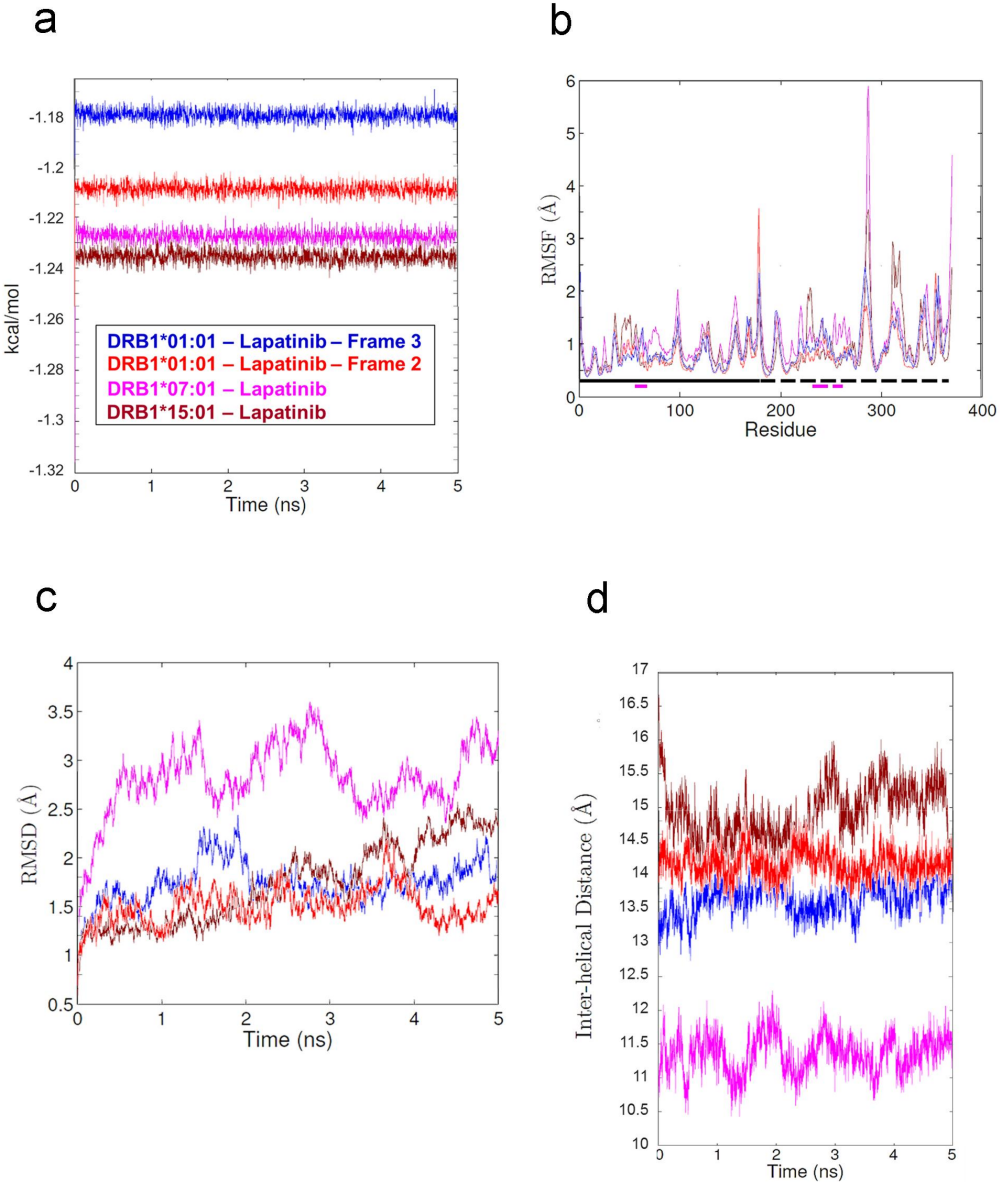
Simulated parameters of trimer HLA system. (a) Calculated Energies vs. time plot. (b) RMSF values of polypeptide backbone. (c) RMSD values of polypeptide backbone vs. time plot. (d) The average inter-helical distances.

**Fig 7:**
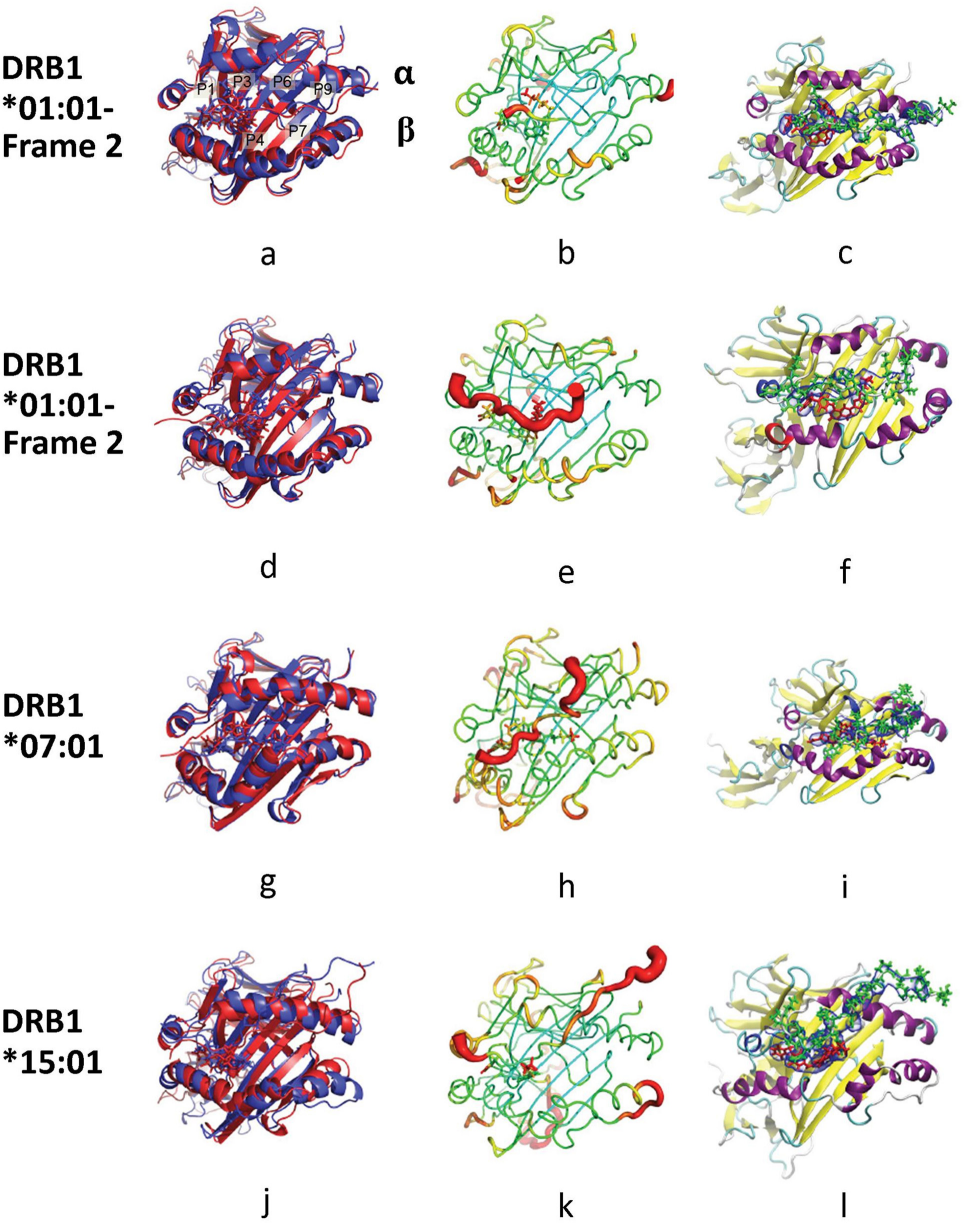
Simulated structures of trimer-HLA system. HLA-DRB1*01:01-Frame 2 (a-c), HLA-DRB1*01:01-Frame 3 (d-f), HLA-DRB1*07:01 (g-i) and HLA-DRB1*15:01 (j-l). (a), (d), (g) and (j): Alignment of initial structure (red) and a representative structure (blue). (b), (e), (h) and (k): Sausage plot of the structure where the color and the thickness of HLA are proportional to the RMSF of α carbon. The color scales for the sausage plots are the same throughout this document. (c), (f), (i) and (l): The volume occupied by lapatinib and peptide. The blue envelope outlines the 50% occupancy volume of the drug (red) and peptide (green).

Each of the trimer simulations is energetically stable (Fig 6a). This is not unexpected because the trimer models were built from the output of the lapatinib-bound HLA and peptide-bound HLA simulations, which all contained stable HLA conformations by the end of the simulations. Although both lapatinib and peptide contribute a relatively small percent of the total system energy and thus their effect is not seen in the graph, the forces applied to each molecule through the MD simulation are still maintained and the compounds move accordingly. Therefore, while we see conformation changes in Fig 7, the energy plot can appear relatively flat. Fig 6b shows that the flexibility of the HLA molecule is very similar in all simulations. Overall, the presence of peptide and lapatinib slightly decreases the overall flexibility of three alleles compared to the apo simulations. Fig 6c shows that the RMSDs stabilize at different rates, depending on the allele and frame. This variability is expected because the different peptides are expected to have different affinities to HLA and lapatinib. Fig 6d shows that the binding groove of the DRB1*07:01 trimer is notably narrower than other systems. In Fig 7, structural analyses of the trimer simulations are presented in three panels. The placement of a peptide lying over the bound lapatinib serves to reduce the mobility and flexibility of lapatinib except for the lapatinib-DRB1*01:01-frame 3 system. Thus, these systems appear more stable in the trimer form than the lapatinib-bound HLA simulations.

The HA peptide frame 2 binds over the top of lapatinib and makes poor contact with P1 of DRB1*01:01. Lapatinib seems to partly bind the P1 pocket and to be relatively stationary (Fig 7a–7c). In frame 3, peptide also binds over the top of lapatinib and its binding to P1-P4 of DRB1*01:01 seems to be disrupted by lapatinib. Lapatinib binding spans P1-P4, but despite starting in the P1 pocket, lapatinib pops out and is less stable than in frame 2 (Fig 7d–7f). Moreover, the integrity of the α chain helix is disrupted in both cases, which could potentially reduce the binding affinities to ligand peptides. With respect to DRB1*07:01, lapatinib lies across the bottom of the binding groove interacting with P1-P6 and possibly descending into P4, which is similar to how lapatinib behaved in the lapatinib-HLA system (Fig 7g–7i). Because lapatinib is bound along a large part of the binding groove, the binding of TT peptide to these pockets seems to be disrupted and indeed TT peptide bends around lapatinib. Similarly to the lapatinib-DRB1*07:01 dimer system, there is a tightly closed binding groove that is not seen in the other three trimers. Despite such a notable conformational change, the integrity of the α chain helix is maintained. With respect to DRB1*15:01, lapatinib lies across the bottom of the binding groove interacting with pockets P1-P4, and does descend into P1 (Fig 7j–7l). This configuration appears to disrupt the MBP peptide binding to P1 and P4 and indeed much of the peptide is not in the groove. Although the integrity of the α chain helix is not disrupted, the α and β chains move significantly.

## Discussion

Idiosyncratic drug toxicity (IDT) is a toxic drug reaction often found independently of drug dose and duration of administration. It occurs rarely, but is a significant cause of morbidity and mortality for patients. Because of its severity and unpredictable nature, it remarkably increases the uncertainty of drug development [13]. Clinical investigations proposed that the mechanisms of most IDTs are immune-mediated and there is a very strong association with specific HLA genes for certain reactions. It has been believed in immunology that small molecules (smaller than 1000 D) are not antigenic in themselves and therefore most of the small molecular drugs would not be immunogenic, which leads to the hapten concept for the cause of IDTs [14]. In recent years, however, another concept known as the “pharmacological interaction with immune receptors” has been reported, claiming that some drugs may directly and reversibly bind to the HLA and/or T cell receptor and stimulate the immune response [15]. Indeed, there have been several reports showing that small molecular compounds called major histocompatibility complex loading enhancers, such as adamantyl derivatives [16] and short peptide fragments [17], can enhance the ligand peptide binding to HLA class II molecules by targeting the P1 pocket of the binding groove. In other case, glyphosine derivatives interact with the P9 pocket and enhance insulin peptide presentation to T cells [18]. Enhancing the peptide presentation would be one of the mechanisms of IDTs, because the immune system could be stimulated by unusual antigen presentation. In fact, it has been reported that abacavir enhances binding of self-peptide LF9 (LSSPVTKSF) to the specific HLA class I molecule HLA-B*57:01 and alters the quantity and quality of self-peptide loaded into HLA-B*57:01 to generate an array of neo-antigen peptides that could drive immune responses leading to toxicity [12]. Our study indicates that lapatinib has a potential to enhance the binding of TT peptide to the HLA-DRB1*07:01 allele, which is associated with lapatinib-induced liver injury, as the first case reporting that the clinically available drug can enhance the binding of ligand peptide to the HLA class II molecule. The finding motivated us to predict the possible interaction mode of lapatinib with HLA-DRs. A series of *in silico* studies indicated that lapatinib lies across the bottom of the binding groove interacting with P1-P4 or P6 and destabilizes these pockets for all three alleles. This mechanism is similar to the case of abacavir. Illing et al. reported that abacavir was bound to HLA-B*57:01 in an extended manner at the base of the peptide binding groove and the presence of abacavir within several binding pockets affects the nature of the peptides bound. For example, P7-Lys of the conventional LF9 peptide would clash with the cyclopentyl and purinyl moieties of abacavir [19]. Considering the enhancing effect of abacavir on the binding of LF9 peptide to HLA-B*57:01, the destabilizations of several pockets by a small molecular drug do not necessarily lead to the displacement of the ligand peptide. Moreover, lapatinib induced notable conformational change in the binding groove, adopting poses where the residues near position 66 in the β chain bend towards the α chain to make a tightly closed binding groove. This notable narrow binding groove was observed only in the lapatinib-DRB1*07:01 system. It is possible that such structural changes are relevant to the binding affinities because changes in the β chain seem to occur in order to make better contact with lapatinib even in the presence of ligand peptide. Therefore, this may be one reason lapatinib enhanced the binding of the ligand peptide to DRB1*07:01 preferentially. The more detailed analysis of the effect by tightly closed binding groove will be an important research subject in the future. Despite such a large conformational change, the integrity of the helix is maintained in the DRB1*07:01 trimer system. Because helix disruption could potentially reduce the binding affinities to ligand peptides, the maintenance of the integrity of helices would be also important.

In summary, our *in vitro* experiments revealed that lapatinib enhances binding of the ligand peptide to HLA-DRB1*07:01 preferentially. Furthermore, our *in silico* modeling supported these findings by showing the allele specific modification of the binding groove of DRB1*07:01. This report provides a new insight into the mechanism of idiosyncratic lapatinib-induced liver injury in the patients who carry the HLA-DRB1*07:01.
